# Changes in neurovascular coupling during cycling exercise measured by multi-distance fNIRS: a comparison between endurance athletes and physically active controls

**DOI:** 10.1007/s00221-019-05646-4

**Published:** 2019-09-10

**Authors:** Oliver Seidel, Daniel Carius, Julia Roediger, Sebastian Rumpf, Patrick Ragert

**Affiliations:** 1 Institute for General Kinesiology and Exercise Science, Faculty of Sport Science, University of Leipzig, Jahnallee 59, 04109 Leipzig, Germany; 2 Department of Neurology, Max Planck Institute for Human Cognitive and Brain Sciences, Leipzig, Germany

**Keywords:** fNIRS, Cycling, Neurovascular coupling, Primary motor cortex, Athletes

## Abstract

It is well known that endurance exercise modulates the cardiovascular, pulmonary, and musculoskeletal system. However, knowledge about its effects on brain function and structure is rather sparse. Hence, the present study aimed to investigate exercise-dependent adaptations in neurovascular coupling to different intensity levels in motor-related brain regions. Moreover, expertise effects between trained endurance athletes (EA) and active control participants (ACP) during a cycling test were investigated using multi-distance functional near-infrared spectroscopy (fNIRS). Initially, participants performed an incremental cycling test (ICT) to assess peak values of power output (PPO) and cardiorespiratory parameters such as oxygen consumption volume (*V*O_2_max) and heart rate (HRmax). In a second session, participants cycled individual intensity levels of 20, 40, and 60% of PPO while measuring cardiorespiratory responses and neurovascular coupling. Our results revealed exercise-induced decreases of deoxygenated hemoglobin (HHb), indicating an increased activation in motor-related brain areas such as primary motor cortex (M1) and premotor cortex (PMC). However, we could not find any differential effects in brain activation between EA and ACP. Future studies should extend this approach using whole-brain configurations and systemic physiological augmented fNIRS measurements, which seems to be of pivotal interest in studies aiming to assess neural activation in a sports-related context.

## Introduction

It is well known that exercise, which is defined as a planned, structured (repetitive) form of distinct physical activities (Budde et al. 2016; Caspersen et al. 1985; Herold et al. 2018b), stimulates cardiovascular, pulmonary and musculoskeletal systems, and its metabolic effects have also been described (Moghetti et al. 2016; Nystoriak and Bhatnagar 2018; Vuori 1995). Contrarily to the profound knowledge about peripheral adaptations to regular exercise, information about its effects on brain function and structure still remains elusive. Recent studies provided compelling evidence that regular exercise and physical activity, which is defined as any bodily, muscle-produced movement (Budde et al. 2016; Caspersen et al. 1985; Herold et al. 2018b), are associated with functional and structural brain alterations (Bullitt et al. 2009; Colcombe et al. 2006; Erickson et al. 2012; Voss et al. 2010; Wagner et al. 2015). On a functional level, Lulic et al. (2017) observed that the propensity for exercise-induced functional plasticity is different in high versus low physically active individuals. Here, a single session of moderate-intensity aerobic exercise increased the amplitude of corticospinal output in the HIGH (physically active) group, and, in contrast, did not alter corticospinal output in the LOW (physically active) group. Furthermore, authors suggest that a single session of aerobic exercise can transiently reduce inhibition in the motor cortex regardless of physical activity level, potentially priming the system for plasticity induction. Apart from causing volume changes (Dordevic et al. 2018; Meier et al. 2016; Weinstein et al. 2012), structural plasticity is associated with changes in cerebral blood flow (CBF) and metabolism (Ide and Secher 2000). Increased metabolic demands due to exercise are met by redistributing oxygen supply in active brain regions from areas that are not necessarily solicited during exercise (Heinonen et al. 2014). With regards to regular physical activity, several cross-sectional studies found that more hours of weekly physical activity and higher cardiorespiratory fitness levels (Albinet et al. 2014; Dupuy et al. 2015) are associated with higher oxyhemoglobin (Hb) levels and superior cognitive performance. However, since physical-activity-induced neurobiological mechanisms are not completely understood yet, it seems reasonable to apply state-of-the-art neuroimaging methods to foster our understanding of the effects of physical activity, exercise and training on neuroplasticity. In general, neuroplasticity can be seen as a phenomenon where the brain adapts its processing as a consequence of, e.g., a changing environment and/or specific demands in everyday life (Pascual-Leone et al. 2005, 2011). Apart from this existing knowledge, the effects of exercise in sport settings still remain elusive.

In the present study, functional near-infrared spectroscopy (fNIRS) was applied in a sport-specific context. This neuroimaging method has been established as a valuable tool for measuring neurovascular coupling (Liao et al. 2013; Pinti et al. 2018) during physical activity and exercise involving the quantification of chromophore concentrations resolved from the measurement of relative changes in oxygenated (Hb) and deoxygenated hemoglobin (HHb) (Obrig et al. 1996; Obrig and Villringer 2003; Perrey 2008; Strangman et al. 2002). Previous studies have demonstrated that fNIRS is an appropriate and reliable method for measuring neurovascular coupling in adults during both simple motor tasks (see Leff et al. 2011) as well as complex body movements such as walking/running (Harada et al. 2009; Holtzer et al. 2011; Suzuki et al. 2004), rowing (Nielsen et al. 1999), squatting (Kenville et al. 2017), juggling (Carius et al. 2016) or playing table tennis (Balardin et al. 2017). Moreover, cycling on an ergometer has been established in several studies as a standardized and comparable task for measuring brain activation during exercise using fNIRS (Ohyanagi et al. 2018; Radel et al. 2018; Tempest and Parfitt 2016; Tempest and Reiss 2018). Nevertheless, previous investigations were mainly limited to fNIRS measurements focusing on prefrontal brain regions (Giles et al. 2014; Jung et al. 2015; Rupp et al. 2008). Our previous knowledge on this topic concerning brain functioning in motor-related areas during cycling is mainly based on studies using positron emission tomography (PET) and electroencephalography (EEG). For example, previous studies using PET (Christensen et al. 2000) and EEG (Brümmer et al. 2011) found that higher motor centers, including M1 and supplementary motor cortices (SMA) as well as cerebellum, take an active part in the generation and control of rhythmic motor tasks such as cycling. These findings were confirmed in fNIRS studies showing increased oxygenation (Takehara et al. 2017) and deoxygenation (Jung et al. 2015) with elevating exercise intensities. Furthermore, previous studies revealed exercise-induced activations in prefrontal cortex (PFC) showing that Hb and HHb levels respond as a function of both exercise load and duration in both experts (Rupp et al. 2008) and non-experts (Auger et al. 2016; Giles et al. 2014; Jung et al. 2015). However, previous studies focusing on neurovascular coupling at several intensity levels show that there is no consensus concerning the type of exercise (incremental tests with short stages vs. constant-load exercises), duration of exercise (Giles et al. 2014; Tsubaki et al. 2016) and intensity (Auger et al. 2016; Byun et al. 2014; Giles et al. 2014; Takehara et al. 2017; Tsubaki et al. 2018).

According to a systematic review by Rooks et al. (2010), exercise-induced responses in neurovascular coupling varied widely between aerobically trained and untrained individuals relating to exercise intensity. At low and moderate intensities, authors suggest a lower cortical metabolic demand in trained people due to lower Hb level and lower blood volume compared to untrained individuals. Contrarily, during maximal and exhaustive intensities, aerobically trained people attained higher levels of cortical Hb, HHb and total hemoglobin than untrained participants. The authors explain these contrasting results by a downregulation of sensory signaling from muscle and limbs to central nervous system (CNS), particularly at submaximal intensities, as an adaption to exercise training (Rooks et al. 2010). As a result, this would lead to a reduced somatosensory input to subcortical and cortical brain regions (Gandevia 2001), and, thus, to a reduced CBF and oxygenation in highly trained individuals, especially at submaximal intensities (Butler et al. 2003; Taylor and Gandevia 2008).

Hence, the present study aimed at (a) revealing intensity-dependent changes in neurovascular coupling in motor-related brain regions and (b) quantifying expertise effects during a short-term constant-load cycling test using a multimodal approach that combined fNIRS and cardiorespiratory measurements. Since fNIRS signal is known to be confounded by extra-cerebral influences (Tachtsidis and Scholkmann 2016), we used a multi-distance fNIRS measurement including multiple short-distance channels to sufficiently control for this matter. The focus was on the question whether a short time window of cycling is capable of inducing alterations in neurovascular coupling at several intensity levels determined by an incremental cycling test (ICT). In general, as a confirmatory hypothesis based on the literature, we expected endurance athletes (EA) to show superior values in peak power output (PPO) and maximum oxygen consumption volume (*V*O_2_max) (Kostić 2017; Rankovic et al. 2010) as assessed by ICT, indicating clear differences in training status as compared to active control participants (ACP). Furthermore, we hypothesized to observe neurovascular coupling alterations (increase in Hb and decrease in HHb) in motor-related brain regions such as M1, SMA and premotor cortex (PMC) in dependence of exercise intensity (Auger et al. 2016; Giles et al. 2014; Kenville et al. 2017) assessed by multi-distance fNIRS measurements during short-term and submaximal constant-load cycling exercises as well as an intensity-dependent increase of cardiorespiratory parameters such as *V*O_2_ and heart rate (HR). Additionally, we assumed, in line with the “neural efficiency” hypothesis (Dunst et al. 2014), that EA would show less neurovascular coupling at distinct intensity levels as compared to ACP. Similar findings have been observed by Ludyga et al. (2016), showing lower brain activation levels in cyclists with high aerobic fitness compared to peers with less aerobic fitness, indicating enhanced neural efficiency in subjects with high aerobic power.

## Materials and methods

### Ethical approval

The study was approved by the local ethics committee of the Medical Faculty at the University of Leipzig. All participants gave written informed consent to participate in the experiments according to the Declaration of Helsinki.

### Participants

In the present study, a total number of 42 healthy, young adults were recruited (Table 1), divided into endurance athletes (EA) and active control participants (ACP). As assessed by the Edinburgh Handedness Inventory (Oldfield 1971), all participants were right-handed. To exclude the presence of any neurological disease and/or contraindications, all participants answered a neurological questionnaire prior to testing phase. Inclusion criteria for EA consisted of an individual training history of at least 2 years and a weekly exercise volume of at least 8 h in their respective sports discipline. ACP were not allowed to do more than 5 h of combined sports activities per week, where no kind of any endurance sports may be included. EA consisted of 17 triathletes, 3 cyclists and 2 cross-country skiers. Additionally, all participants with regular practice of musical instruments were excluded from participation since recent studies have shown that musical training induces functional and structural plasticity in motor-related brain regions (Steele et al. 2013; Vollmann et al. 2014) which in turn might affect the assessment of neurovascular coupling.

**Table 1:** Participant demographics

	Endurance athletes (EA)	Active control participants (ACP)
*n* (male/female)	22 (14/8)	20 (12/8)
Mean age [years]	28.82 ± 3.92*	24.80 ± 3.14
Body height [m]	1.77 ± 0.10	1.76 ± 0.09
Body weight [kg]	69.55 ± 10.10	69.90 ± 12.08
Training age [years]	7.14 ± 4.82	
Exercise volume [h/week]	11.00 ± 3.99*	3.03 ± 2.78
Laterality quotient (LQ)	85.69 ± 22.23	88.57 ± 16.02

Values are mean ± SD

*Indicates significant differences between EA and ACP (*p* < 0.05)

### Experimental design

**Fig. 1:**
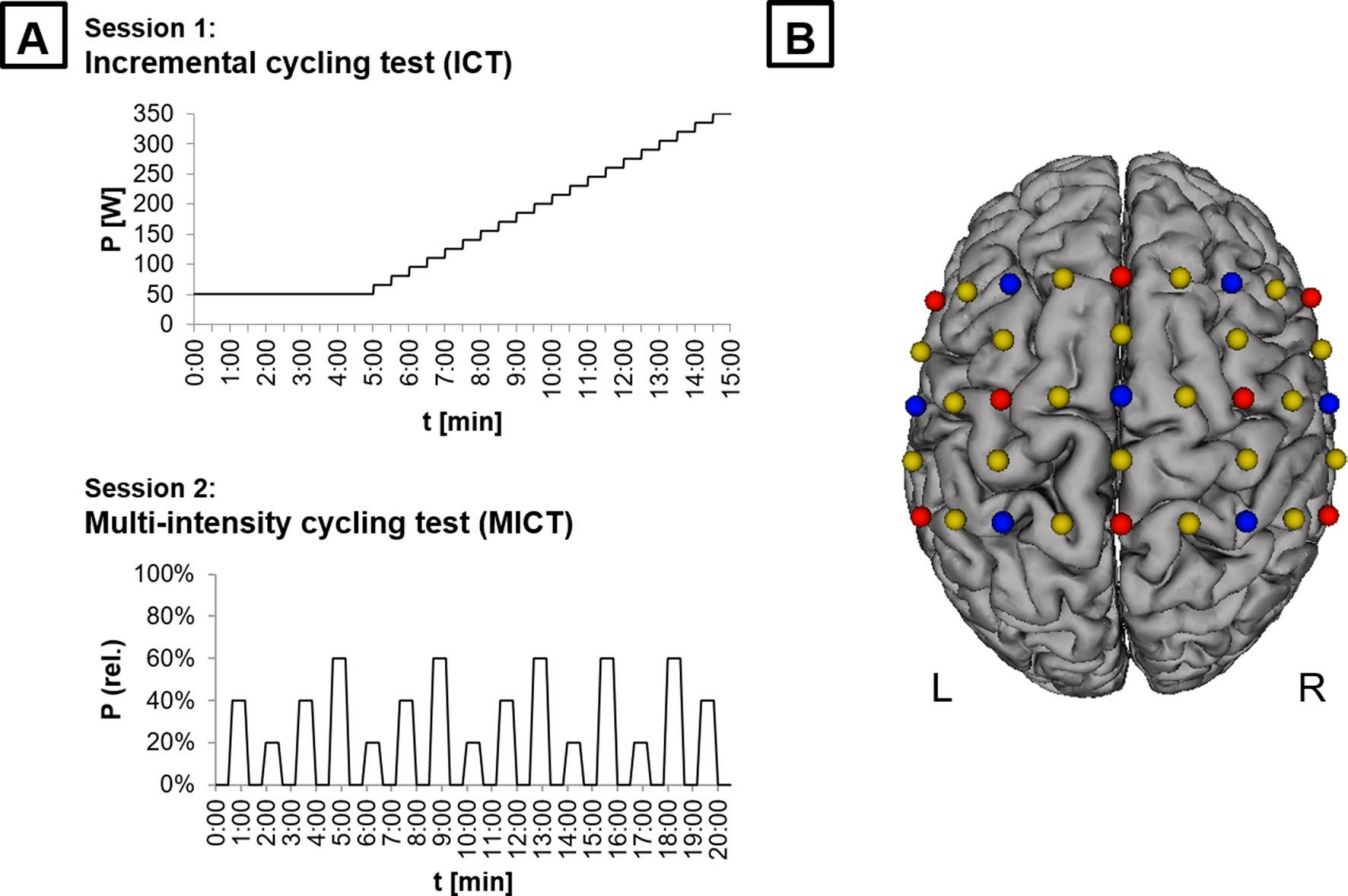
Study design and experimental setup. **a** Procedures for session 1 and 2. The first session consisted of an incremental cycling test (ICT) starting with a 5 min warm-up phase using a constant load of 50 W. Until voluntary exhaustion, participants pedaled continuously with a pedaling frequency of 60–80 revolutions per minute (rpm) during 30-s levels, with each level providing an increment of 15 W in resistance. Using peak power output (PPO), three individual intensity levels of 20, 40, and 60% of PPO were derived that were cycled multiple times during session 2 [multi-intensity cycling test (MICT)]. During MICT, participants cycled 5 trials of 30 s each, followed by a 30-s phase of rest after each activity phase. **b** Illustration of fNIRS configuration used during MICT. Transmitters are shown as red dots and detectors as blue dots. Yellow dots represent the centers of the 22 long (standard) channels. Additionally, eight short-distance detectors (not included in the figure) for each source with an inter-optode distance of 8 mm were used, as opposed to the inter-optode distance for all other (long) channels of our configuration (3 cm)

We used a quasi-experimental design where each participant was tested in two sessions (Fig. 1a) that were separated by at least 24 h to avoid task-related impacts of cognitive or muscular fatigue. Participants were instructed to avoid alcohol and caffeine 24 h prior to each session due to their influences on motor control and CNS functioning (Pesta et al. 2013). Additionally, participants were asked to report their daily activities 48 h prior to each session, along with their current levels of attention, fatigue and discomfort on a visual analog scale (pre and post), as well as their individual amount of sleep the night before experimental sessions.

The first session consisted of an incremental cycling test (ICT) to assess PPO, *V*O_2_max and HRmax of each participant. Using these values, three individual intensity levels of 20, 40, and 60% of PPO were derived. During session 2, participants cycled these levels multiple times while measuring cardiorespiratory responses and neurovascular coupling in motor-related brain regions.

### Motor tasks

A high-performance stationary bicycle (Cyclus 2, RBM elektronik-automation GmbH, Leipzig, Germany) allowing participants to cycle at a constant load measured in watts (W) was used to determine *V*O_2_max and its corresponding load. Before each session, the cycle ergometer was individually adjusted to the participant’s anthropometry.

#### Session 1: Incremental cycling test (ICT)

After adjusting the cycle ergometer and applying the spirometric system including facemask for *V*O_2_ and chest strap for HR, session 1 started with a 5 min warm-up phase using a constant load of 50 W. This load was also the starting point for ICT that started immediately afterwards. Until voluntary exhaustion, participants pedaled continuously with a pedaling frequency of 60–80 revolutions per minute (rpm) during 30-s levels, each level providing an increment of 15 W in resistance (Fig. 1a). Ramp tests like this are commonly used to assess individual PPO and *V*O_2_max quickly and efficiently (Barker et al. 2011; Chicharro et al. 1997). PPO for each participant was determined by the power of the last completed level. The exercise was considered to be maximal when the following criteria were obtained: no change in *V*O_2_ while increasing resistance, participants signal subjective (maximal) exhaustion, or the inability of participants to maintain pedaling frequency despite maximum effort. After ICT was stopped, resistance was reduced to zero, and participants were asked to continue unloaded pedaling for another 4–5 min until physiological parameters nearly reached warm-up values.

#### Session 2: Multi-intensity cycling test (MICT)

In preparation for session 2, a fNIRS cap was placed on the participant’s head in addition to the spirometric system. After all devices were calibrated and baseline measurement was completed, participants performed a 5 min warm-up phase using a constant load of 50 W. Subsequently, using a block design, participants cycled five trials of 30 s each at intensities of 20, 40, and 60%, respectively (Fig. 1a). The order of trials was randomized, with the exception of avoiding more than two consecutive trials of one intensity. Similar to session 1, participants were instructed to maintain a cycling rate of 60–80 rpm. Each trial was followed by a 30-s resting phase, where participants were asked to sit completely still on the cycle ergometer. Moreover, participants were asked to avoid upper body and head movements during the whole test to prevent fNIRS signal from artifacts. Further artifacts are due to systemic influences such as an increased blood pressure which has been shown during (static) resistance exercises (MacDougall et al. 1985, 1992) but also during cycling on an ergometer (Kim et al. 2013). Hence, before and after each activity phase, we implemented a 10-s transition phase where participants were supposed to gradually start and stop pedaling, respectively. Using this phase, we avoided a critical systemic influence on the fNIRS signal, especially while initiating the pedaling. Consequently, we measured a pure 30-s phase of activity and rest, respectively.

### Functional near-infrared spectroscopy (fNIRS)

Hemodynamic response alterations during MICT were assessed using the portable NIRSport measuring system (NIRx Medical Technologies, Glen Head, NY). For this purpose, a cap with a defined number of optodes, which were placed directly on the scalp, was applied on the participant’s head. The center of the cap was placed according to the international 10–20 system over vertex (Cz), determined over the intersection of the courses nasion to inion and left to right pre-auricular point (Jurcak et al. 2007). The arrangement of the optodes allowed the measurement of brain activity within human motor system using a fNIRS configuration with a total number of 15 optodes (8 sources and 7 detectors, Fig. 1b). The aim of using this distinct cap configuration was motivated by the fact that we intended to disentangle hemodynamic response alterations as a function of applied intensities, specifically within the bilateral sensorimotor system, including cortical motor regions such as M1, PMC, SMA, superior parietal lobe (SPL) and inferior parietal lobe (IPL). Infrared light was emitted by sources with wavelengths of 760 and 850 nm (Pereira et al. 2007) and measured with a recording frequency of 7.81 Hz. Spectroscopically, NIRSport operated via a so-called continuous wave method; i.e., sources emit light at constant frequency and intensity (Scholkmann et al. 2014).

In addition to the 22 long-distance (standard) channels, we used a short-distance detector bundle (NIRx Medical Technologies, Glen Head, NY) to eliminate potential fNIRS confounders, such as extra-cerebral blood flow alterations (Huppert et al. 2009; Tachtsidis and Scholkmann 2016). For this purpose, we used additional short-distance detectors for each source with an inter-optode distance of 8 mm, as opposed to the inter-optode distance for all other (long) channels of our configuration (3 cm). This resulted in eight short-distance channels, which were considered in the analysis of fNIRS signal (see “Analysis”).

### Cardiorespiratory measurement

Cardiorespiratory parameters were assessed using the Metamax 3B (MM3B) system (Cortex Biophysik GmbH, Leipzig, Germany), which has previously been tested as a stable and reliable gas analysis system (Macfarlane and Wong 2012). MM3B is a portable metabolic system composed of a measurement module and a battery module which can be placed on the participant’s upper chest or back using connecting cords and assisting straps. The breath-by-breath system measures volume of inspired and expired air using a bidirectional digital turbine inserted into the facemask. Parameters of ventilation such as *V*O_2_ were calculated using standard metabolic algorithms (Wasserman 2012). Measurements were performed using Metasoft 3 software, version 3.7.0 SR2.

Prior to using, the system was switched on for at least 20 min, and then calibrated prior to each test according to the manufacturer’s recommendations. This involved calibrating the gas analysers using a reference gas (Hong Kong Specialty Gases), and then verifying the calibration against ambient air. Second, volume calibration was performed using a standardized 3 l syringe (5530 series, Hans Rudolph, Inc., MO, USA).

Once all preparations have been completed, the appropriate facemask was selected for each participant. Covering nose and mouth, the facemask was secured via an adjustable nylon harness and placed over the fNIRS cap without any disturbances of optode channels. Additionally, a chest strap (Polar Electro Oy, Kempele, Finnland) was applied to assess HR. Subsequently, participants were asked to sit completely still for ~ 1 min to perform baseline measurements.

### Analysis

#### Hemodynamics

fNIRS data analysis was performed in MATLAB (MathWorks, Natick, MA, USA) using functions provided in HOMER2 package (Huppert et al. 2009). Statistical analysis was performed using R 3.4.3 (R Core Team, 2017) and RStudio 1.1.383 (RStudio Team, 2017). As a first step, the signal quality of all individual channels was checked by means of a coefficient of variation (CV, exclusion value: 15%). Subsequently, raw fNIRS recordings were pre-processed to reduce the influence of motion artifacts and physiological noise before estimating changes in concentrations of Hb and HHb. First, raw-intensity signals were converted to changes in optical density (Huppert et al. 2009). Correction for motion artifacts was performed using wavelet filtering, which has been described as a promising approach to reduce the influence of motion artifacts in fNIRS recordings (Brigadoi et al. 2014). We used an algorithm described by Molavi and Dumont (2012), as implemented in HOMER2 hmrMotionCorrectWavelet filtering function. The algorithm applies a probability threshold to remove outlying wavelet coefficients, which were assumed to correspond to motion artifacts. We used a threshold of 1.219 times the inter-quartile range (Wiggins and Hartley 2015). Subsequently, data were band-pass filtered to attenuate low-frequency drift and cardiac oscillations using 0.01 Hz as high and 0.5 Hz as low-pass cutoff frequencies (Huppert et al. 2009). Attenuation changes of both wavelengths (850 nm and 760 nm) were transformed to concentration changes of Hb and HHb using the modified Beer–Lambert approach [partial path length factor: 6.0 (Huppert et al. 2009)]. Reporting both (Hb and HHb) changes instead of only one of them is strongly recommended and allows better physiological interpretation of functional experimental results (Tachtsidis and Scholkmann 2016). In general, HHb is described as the more valid parameter for the investigation of hemodynamic response alterations (Muthalib et al. 2015; Piper et al. 2014). Hb signals are more strongly affected by global processes in both extra-cerebral and intra-cerebral compartments and local scalp blood flow regulation, while HHb signals are less contaminated by extra-cerebral processes (Kirilina et al. 2012). To regress extra-cerebral contaminations (measured by short-distance channels) out of the signal, we used HOMER2 hrmDeconvHRF_DriftSS function as previously applied by (Aasted et al. 2016). As implemented in this function, short separation regression (SSR) is performed with the nearest short separation channel while simultaneously estimating HRF (Gagnon et al. 2011) using ordinary least squares (Ye et al. 2009) and a consecutive sequence of Gaussian functions. This method assumes that the signal measured by short-distance channels represents superficial layers and the signal measured by long-distance channels represents both brain tissue and superficial layers. Thus, the effects of systemic physiology can be captured from superficial layers and can then be used as regressors to filter systemic interference from long-distance channels to provide a more robust estimation of underlying hemodynamic alterations to brain activation (Yücel et al. 2015). Afterwards, single trials were baseline-corrected (regarding last 5 s of previous resting phase) and time courses of Hb and HHb concentration changes in each channel were block-averaged using HOMER2 hmrBlockAvg function. Though the experimental block design included long breaks to prevent overlapping of hemodynamic responses between trials, we directly analyzed the height of amplitude [baseline-corrected average of 30-s temporal window with regard to stimulus onset for activity phase (Herold et al. 2018a)].

In fNIRS studies, assumptions for parametric tests are often violated (e.g., normal distribution), as it was for data assessed in the present study. For this reason, robust statistical methods (Wilcox 2017) are a considerable option for data analysis as suggested by Santosa et al. (2018). Hence, differences in Hb and HHb concentration changes between EA and ACP (group as between-subject factor), respectively, intensity levels of 20, 40 and 60% (condition as within-subject-factor) were tested using a robust two-way factorial ANOVA. Additionally, robust dependent *t* tests were conducted on a group level in a channel-wise manner to test contrasts of activity phases against zero (Piper et al. 2014). These tests were conducted in R using WRS2 package (Mair and Wilcox 2017). As a measure of effect size, Wilcox and Tian (2011) proposed an explanatory measure of effect size *ξ*. Values of *ξ* = 0.10, 0.30, and 0.50 correspond to small, medium, and large effect sizes. To control for multiple comparisons during robust *t* tests and ANOVA, we applied false discovery rate (FDR) correction (Singh and Dan 2006). For all tests, critical level of significance was set to *p* < 0.05 and FDR-adjusted for multiple comparisons. The resulting channel-wise *t* values, respectively, *f* values were mapped using the Brain Function Mapping Tool (Wang et al. 2016). To assign hemodynamic response alterations during MICT to a specific brain region, we utilized a probabilistic atlas implemented in the software fOLD [fNIRS Optodes’ Location Decider (Zimeo Morais et al. 2018)] using the automated anatomical labeling atlas AAL2 (Rolls et al. 2015).

#### Cardiorespiratory responses

Similar to fNIRS data, statistical analysis was performed using R 3.4.3 (R Core Team, 2017) and RStudio 1.1.383 (RStudio Team, 2017). Regarding cardiorespiratory measurements during ICT, maximum values of *V*O_2_ and HR were output directly from Metasoft 3. First, absolute values of *V*O_2_ and PPO were calculated in relation to the participant’s body weight to make them comparable inter-individually. Univariate analyses of variance (uANOVA) with factor group (EA vs. ACP) were conducted to test differences between EA and ACP in PPO, *V*O_2_max and HRmax. Partial eta-squares (*η*_p_^2^) for uANOVAs are provided as measures of effect size and used to aid in interpretation of inferential statistics. As a rule of thumb, *η*_p_^2^ ≥ 0.01 is considered to be a small, *η*_p_^2^ ≥ 0.06 a medium, and *η*_p_^2^ ≥ 0.14 a large effect (Miles and Shevlin 2000).

Regarding MICT, intensity-dependent changes in *V*O_2_ and HR were calculated as differences (Δ) between averaged last 5 s of each activity phase (due to delay of the respiratory system during exercise (Roston et al. 1987; Whipp 1994)) and averaged last 5 s of the corresponding previous resting phase (according to processing of fNIRS data). Similar to fNIRS data, differences in Δ*V*O_2_ and ΔHR between EA and ACP (group as between-subject factor), respectively, intensity levels of 20, 40, and 60% (condition as within-subject-factor) were tested using a two-way factorial ANOVA.

Using WRS2 package in R, robust correlation coefficients were calculated to correlate channel-wise intensity-dependent neurovascular coupling changes of Hb and HHb with maximum values from ICT (PPO, *V*O_2_max, HRmax) and with intensity-dependent cardiorespiratory responses (Δ*V*O_2_ and ΔHR). For all tests, a *p* value of < 0.05 was considered significant and FDR correction was applied for multiple comparisons.

## Results

### ICT performance

**Fig. 2:**
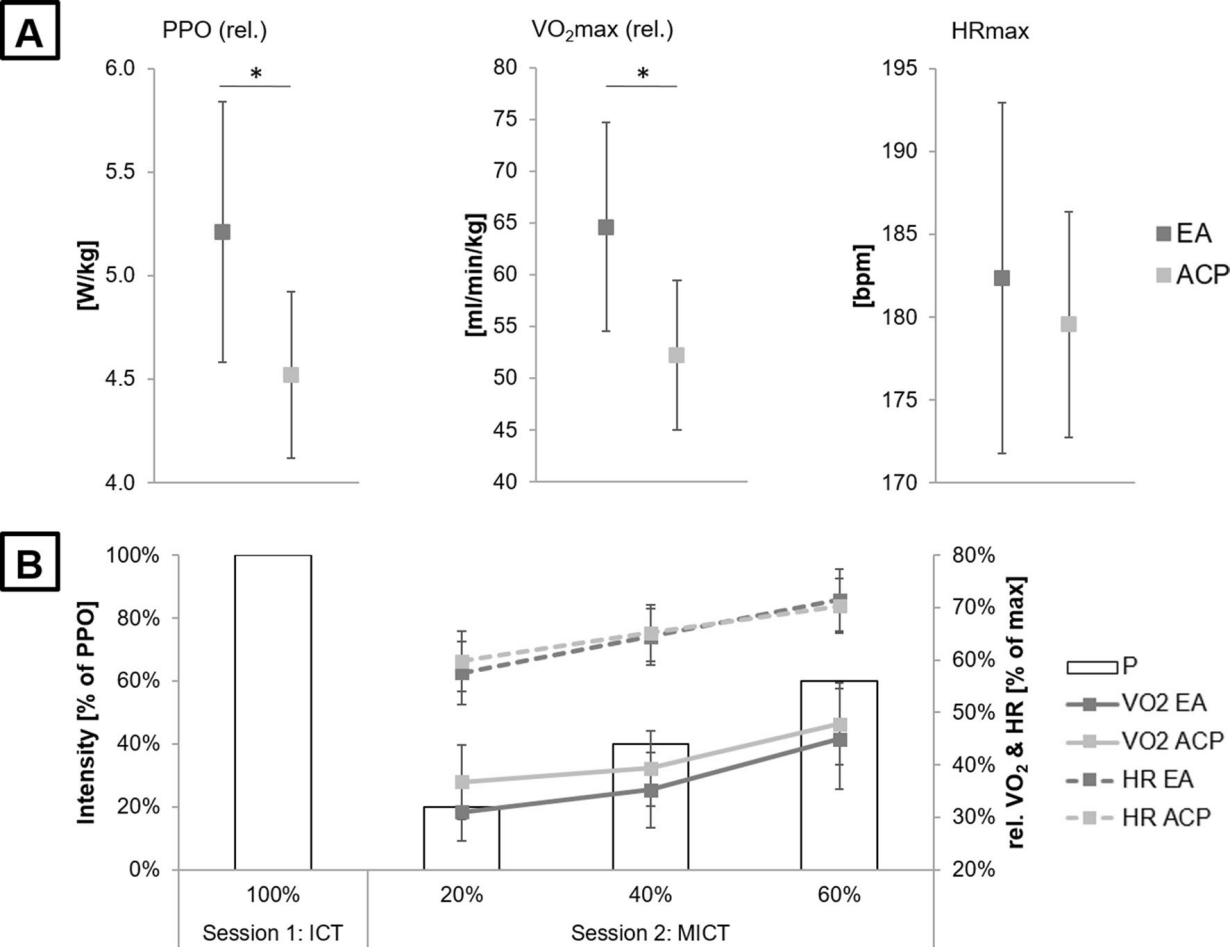
ICT and MICT results. **a** Peak values of power output (PPO), oxygen consumption volume (*V*O_2_max) and heart rate (HRmax) as assessed by incremental cycling test (ICT). Values are mean ± SD; dark gray dots represent endurance athletes (EA), and light gray dots represent active control participants (ACP). *p < 0.5 indicates significantly better PPO and *V*O_2_max values for EA as compared to ACP. **b** Relative values of *V*O_2_ and HR during multi-intensity cycling test (MICT). Values are mean ± SD; dark gray lines represent EA, and light gray lines represent ACP. All values of *V*O_2_ and HR are normalized to peak values (= 100%) as assessed by ICT. Black frames represent PPO as assessed by ICT and three individual intensity levels of 20, 40, and 60% of PPO that were cycled multiple times during MICT

ICT revealed significant differences between EA and ACP in PPO, *V*O_2_max and HRmax. uANOVA revealed significantly higher PPO (EA: 5.21 ± 0.63 W/kg vs. ACP: 4.52 ± 0.40 W/kg; main effect of group: *F*_(1,40)_ = 17.421, *p* < 0.000, *η*_p_^2^ = 0.303, 95% CI [0.355, 1.022]) and *V*O_2_max (EA: 64.59 ± 10.07 ml/min/kg vs. ACP: 52.20 ± 7.21 ml/min/kg; main effect of group: *F*_(1,40)_ = 20.658, *p* < 0.000, *η*_p_^2^ = 0.341, 95% CI [6.881, 17.901]) values in EA as compared to ACP, indicating clear differences in cardiorespiratory fitness level between groups (Fig. 2a). No differences were found in HRmax (EA: 182.36 ± 10.59 bpm vs. ACP: 179.55 ± 6.83 bpm; main effect of group: *F*_(1,40)_ = 1.024, *p* = 0.318, *η*_p_^2^ = 0.025, 95% CI [− 2.806, 8.434]).

### Intensity-dependent changes in neurovascular coupling

**Fig. 3:**
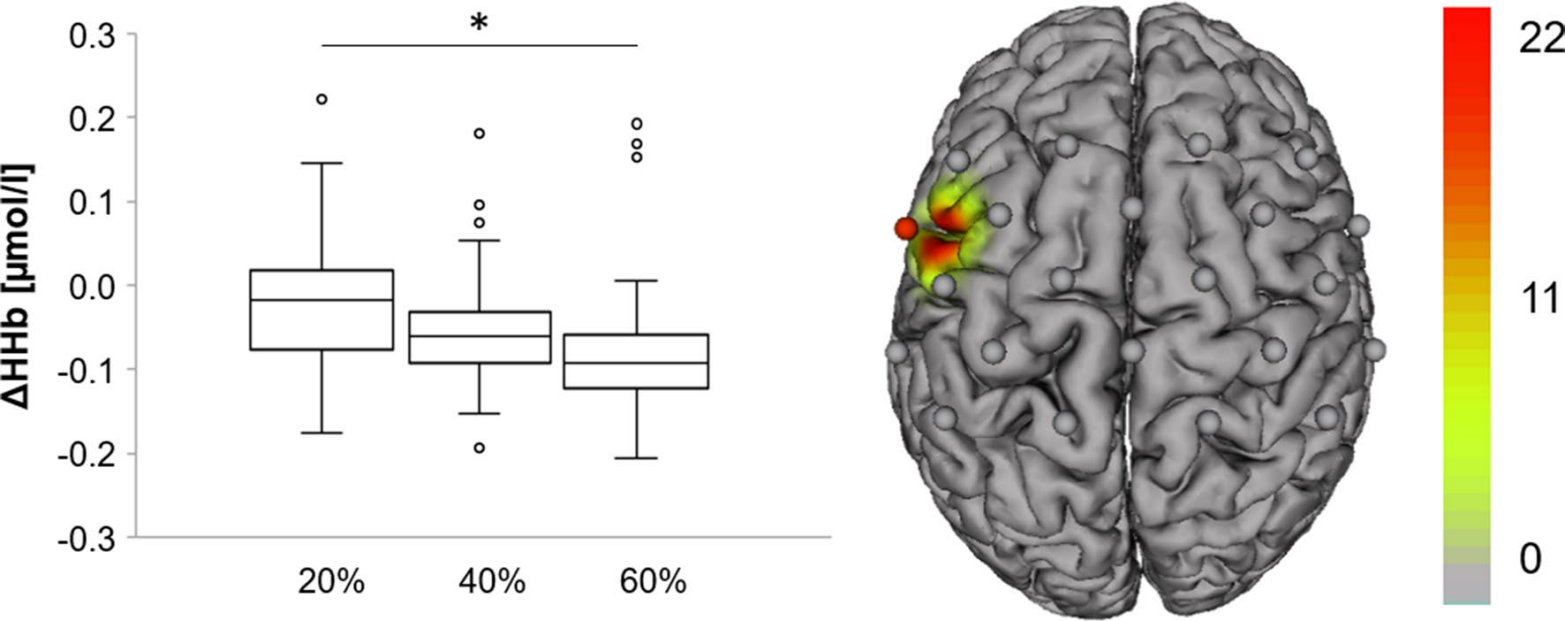
Intensity effect on fNIRS signal during MICT after short-separation regression (SSR). Boxplots represent all participants (EA and ACP). Values are median and interquartile range (being the 25th and 75th percentile). Channels (centered between transmitters and detectors) are shown for the topographic image (*L* left hemisphere, *R* right hemisphere); colors represent *f* values. Images are thresholded at *p* < 0.05 and FDR-corrected for multiple comparisons. *p < 0.5 indicates a significant influence of factor condition on HHb concentration (*F*_(2,80)_ = 18.049, *p*_adjusted_ = 0.001, *ξ* = 0.382), indicating a larger HHb decrease across all participants at an intensity of 60% as compared to 20% (*p*_adjusted_ < 0.000) in left premotor cortex (PMC)

In all channels tested, we did not find any condition x group interaction during MICT neither for Hb (0.014 ≤ *F*_(2,80)_ ≤ 1.688, 0.444 ≤ *p*_adjusted_ ≤ 0.980, 0.012 ≤ *ξ* ≤ 0.107) nor HHb (0.014 ≤ *F*_(2,80)_ ≤ 1.662, 0.462 ≤ *p*_adjusted_ ≤ 0.993, 0.007 ≤ *ξ* ≤ 0.121). However, in left PMC, we found a significant influence of factor condition on HHb concentration (*F*_(2,80)_ = 18.049, *p*_adjusted_ = 0.001, *ξ* = 0.382, 95% CIs [− 0.001, 0.060], [− 0.008, 0.156], [0.235, 0.148]), indicating a larger HHb decrease across all participants at an intensity of 60% as compared to 20% (*p*_adjusted_ < 0.000) (Fig. 3).

**Fig. 4:**
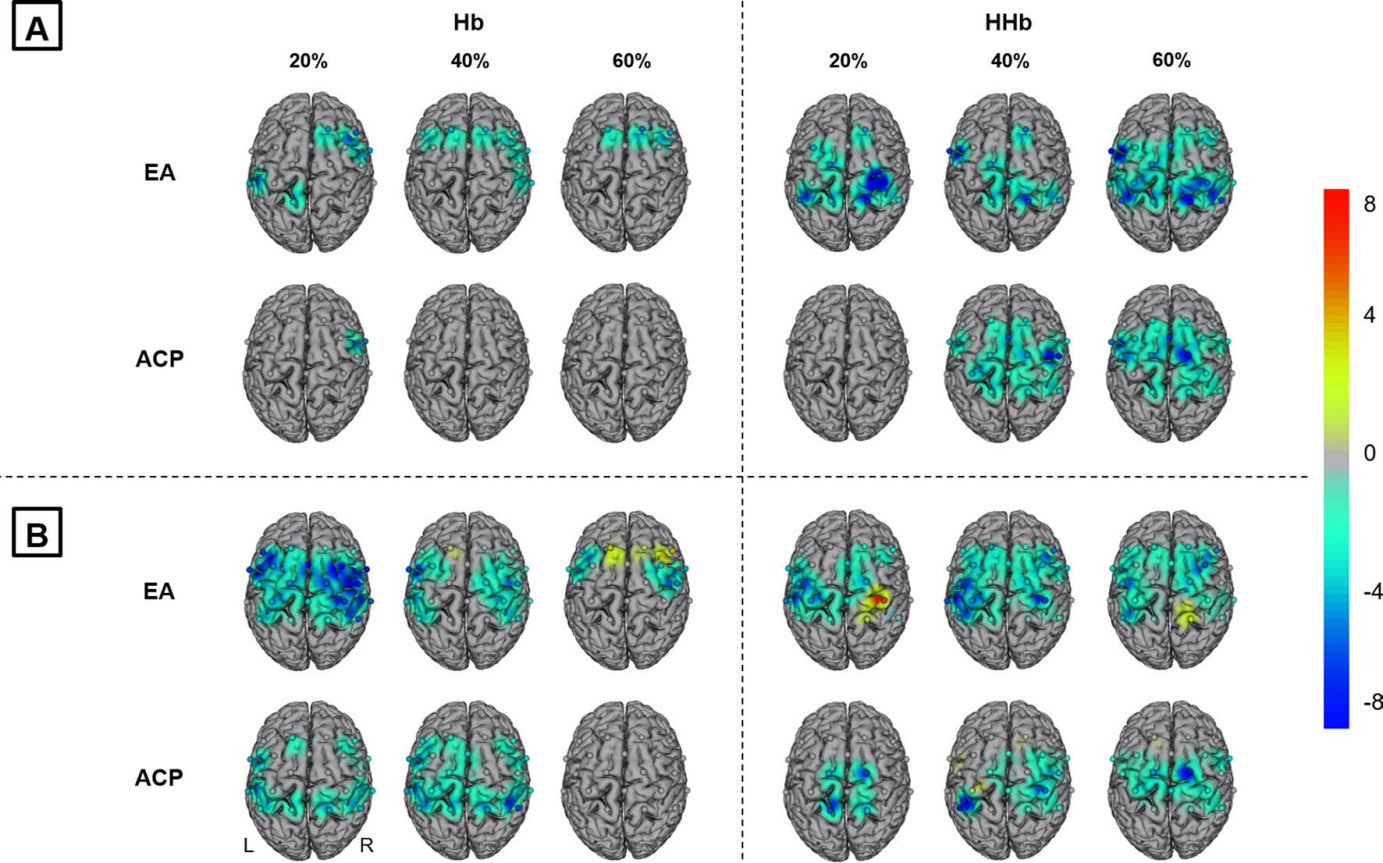
Hemodynamic response alterations during MICT. Channels (centered between transmitters and detectors) are shown for each image (*L* left hemisphere, *R* right hemisphere). Decreases are illustrated in dark blue and increases in dark red; colors represent t-values. All images are thresholded at *p* < 0.05 and FDR-corrected for multiple comparisons. Topographic images show significant decreases of both Hb and HHb. **a** Within-group comparisons for Hb and HHb. Comparisons were performed using robust dependent *t* tests testing activity phases (20, 40, and 60% of PPO) of endurance athletes (EA) and active control participants (ACP) against zero revealing significant decreases of Hb and HHb in both groups. **b** Effect of short-separation regression (SSR). Tests were performed before and after SSR to compare the effect of short-distance channels. The image illustrates the deltas between alterations in brain activation before and after SSR, indicating extra-cerebral signals and systemic interferences that were regressed out of the long-distance channels by SSR

In a further analysis step at a group level, we aimed to illustrate hemodynamic response alterations for each intensity level separately. Robust independent *t* tests revealed significant decreases of Hb and HHb in both groups (Fig. 4a). We found decreased Hb concentrations in precentral areas such as PMC and SMA and left-hemispheric IPL for EA and only in right-hemispheric PMC (at 20%) for ACP. HHb concentrations decreased bilaterally in pre- and postcentral areas as well as in M1 in both groups, except ACP at 20% showing no significant HHb alterations.

Additionally, robust independent *t* tests were also performed without applying SSR. Figure 4b elucidates deltas between alterations in brain activation before and after SSR, indicating that hrmDeconvHRF_DriftSS function was capable of filtering systemic interferences from long-distance channels.

### Intensity-dependent cardiorespiratory responses

During MICT, robust two-way factorial ANOVA revealed significant influences of factors condition (*F*_(2,80)_ = 63.450, *p*_adjusted_ = 0.001, *ξ* = 0.401, 95% CIs [− 9.140, − 20.448], [− 16.768, − 0.159], [− 10.572, − 4.954]) and group (*F*_(2,80)_ = 5.916, *p*_adjusted_ = 0.019, *ξ* = 0.254, 95% CI [1.077, 11.197]) on *V*O_2_, but a non-significant condition x group interaction (*F*_(2,80)_ = 4.307, *p*_adjusted_ = 0.134, *ξ* = 0.101, 95% CIs [− 5.592, − 8.994], [− 8.862, 3.389], [0.882, 2.952]) (Fig. 2b). Post-hoc tests revealed significant differences between all intensity levels (20 vs. 40%: *p*_adjusted_ = 0.014; 20 vs. 60%: *p*_adjusted_ < 0.000; 40 vs. 60%: *p*_adjusted_ < 0.000), indicating intensity-dependent increase in *V*O_2_.

Concerning intensity-dependent HR changes during MICT, robust two-way factorial ANOVA revealed significant influences of factors condition (*F*_(2,80)_ = 170.639, *p*_adjusted_ = 0.001, *ξ* = 0.432, 95% CIs [− 29.599, − 52.907], [− 31.148, − 14.596], [− 35.763, − 13.327]) and group (*F*_(2,80)_ = 16.944, *p*_adjusted_ = 0.001, *ξ* = 0.398, 95% CI [8.754, 25.242]), but a non-significant condition x group interaction (*F*_(2,80)_ = 4.076, *p*_adjusted_ = 0.146, *ξ* = 0.127, 95% CIs [− 10.774, − 15.458], [− 12.524, 4.229], [1.686, 5.297]) (Fig. 2b). Post-hoc tests revealed significant differences between all intensity levels (20 vs. 40%: *p*_adjusted_ < 0.000; 20 vs. 60%: *p*_adjusted_ < 0.000; 40 vs. 60%: *p*_adjusted_ < 0.000), indicating intensity-dependent increase in HR.

### Correlation analysis

**Fig. 5:**
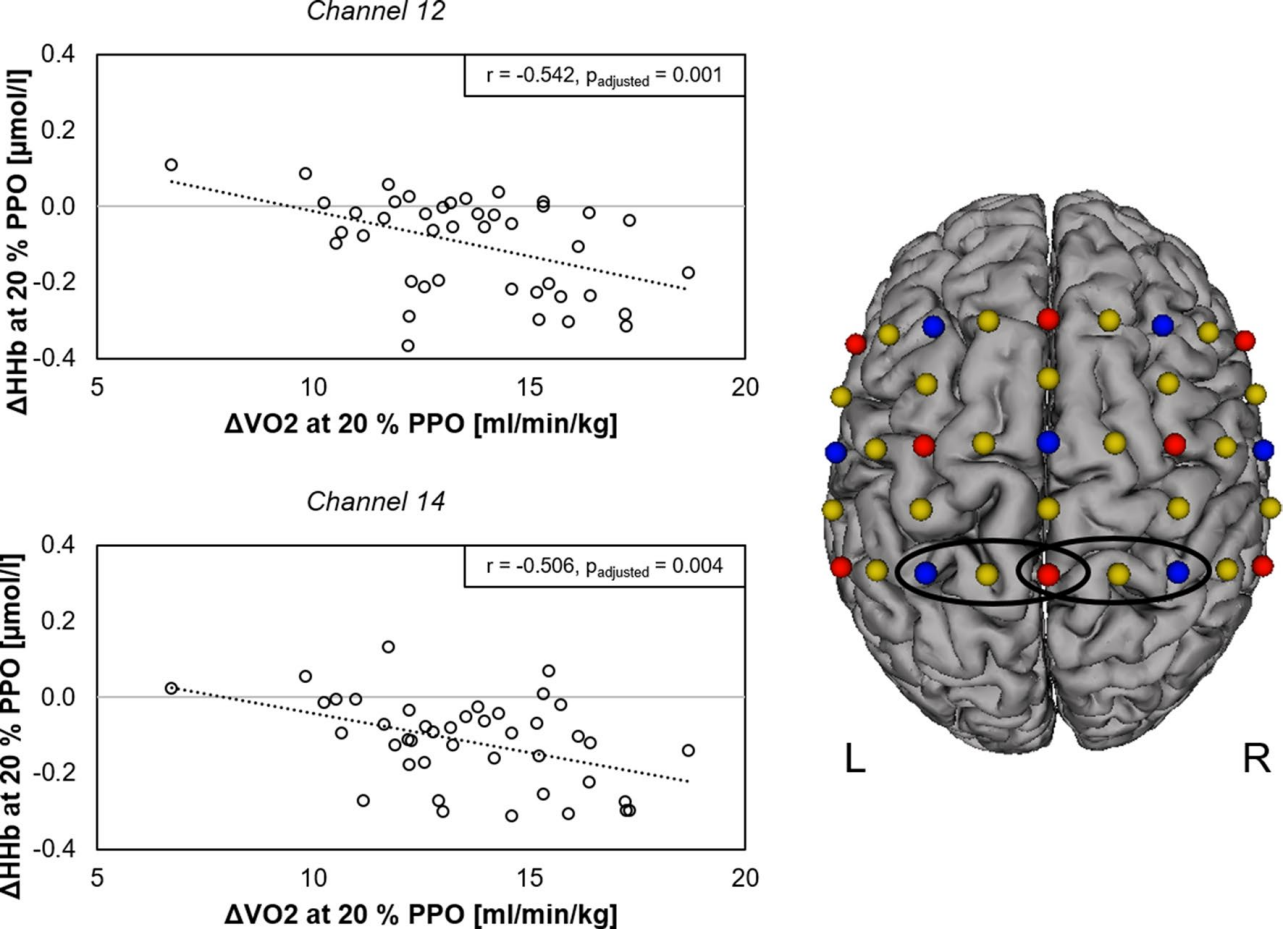
Correlation analysis between intensity-dependent HHb alterations and *V*O_2_ changes at 20% of PPO. Scatter plots for all participants (EA and ACP) show significant negative associations within bilateral superior parietal lobe for HHb in two channels (*r* = − 0.542, *p*_adjusted_ = 0.001 and *r* = − 0.506, *p*_adjusted_ = 0.004). ΔHHb and Δ*V*O_2_ are the differences between activity at 20% of PPO and the previous resting phase. fNIRS configuration on the right shows the channels of the significant negative associations (black circles). Transmitters are shown as red dots and detectors as blue dots. Yellow dots represent the centers of the 22 long (standard) channels

Robust correlation analysis revealed significant negative correlations in two channels (*r* = − 0.542, *p*_adjusted_ = 0.001 and *r* = − 0.506, *p*_adjusted_ = 0.004) between HHb concentration changes and Δ*V*O_2_ at an intensity of 20% in bilateral SPL (Fig. 5). These findings indicate that larger intensity-dependent increases of VO_2_ are correlated with larger decreases of HHb at the lowest intensity level. All other correlation analyses concerning intensity-dependent alterations and peak values of measured parameters revealed no further significant results.

## Discussion

The present study aimed to investigate whether a short-term cycling exercise is capable of inducing intensity-dependent hemodynamic response alterations in motor-related brain regions. Using a multimodal approach that combined multi-distance fNIRS and cardiorespiratory measurements, trained endurance athletes (EA) as well as active control participants (ACP) were tested to reveal possible expertise effects. The study focused on the question whether neurovascular coupling depends on exercise intensity and if EA would differ in their exercise-induced brain activation compared to ACP. Our results provide novel evidence concerning the practicability of fNIRS measurements including multiple short-distance channels during a cycling task. We found typical exercise-induced alterations in the more valid parameter HHb as well as a correlation between HHb concentration changes and *V*O_2_ at the lowest intensity level. However, our findings suggest that the “neural efficiency” hypothesis (Dunst et al. 2014) seems not to be applicable for highly automated movements such as cycling on an ergometer.

### Hemodynamic and cardiorespiratory responses during MICT

We hypothesized to observe an increase in Hb and simultaneously a decrease in HHb in motor-related brain regions in dependence of exercise intensity as well as intensity-dependent increases of cardiorespiratory parameters such as *V*O_2_ and HR. This assumption was confirmed by all parameters except Hb. As well known, changes in regional brain activation in response to motor stimulation are associated with higher concentrations of Hb and decreased concentrations of HHb (Obrig et al. 1996; Obrig and Villringer 2003; Perrey 2008; Strangman et al. 2002). Contrarily to this evidence, we found exercise-induced decreases of Hb particularly in precentral brain regions of EA. Such atypical findings were also found in a recent study by Takehara et al. (2017). In this study, participants performed 10 min of exercise at intensities of 30 and 50% of *V*O_2_max on a cycle ergometer while Hb concentrations were measured in PFC and M1. Interestingly, they found that Hb concentrations were significantly decreased in the initial phase (first 2–3 min) of exercise in both regions of interest, while they were significantly increased from mid to final phase for both intensities compared with baseline values. Since our activity phase of 30 s coincides with the initial phase of this study, both observations are similar and comparable. According to the authors, the initial phase of complex movements might be a period during which oxygen supply is insufficient for oxygen demands of the brain. As previously shown, neural activity increases in response to increased tissue oxygen consumption and glucose metabolism and thus, leading to an increased blood flow in the brain (Fox et al. 1988). These increases in CBF can be observed approximately 3 s after an increase in neural activity in the brain (Bandettini et al. 1992), whereupon changes are observed at intervals of about 6 s until sufficient increases have occurred (Taoka et al. 1998). During this time, a transient decrease in Hb concentrations can be observed (Takehara et al. 2017). This phenomenon was underlined by a recent study where participants performed 15-min cycling exercises at intensities of 20, 40, and 60% of PPO (Endo et al. 2013). During ergometer exercise, Hb concentrations in PFC initially decreased until 5 min from onset of exercise and increased during later periods. Moreover, initial decreases and later increases of Hb were even dependent on exercise intensity.

To summarize, changes in Hb are considered to be a more sensitive indicator of global processes in both extra-cerebral and intra-cerebral compartments and local scalp blood flow regulation than changes in HHb, while changes in HHb are determined more by venous oxygenation and blood volume than blood flow (Hoshi et al. 2001). These facts resulted in current discussions about reliability and validity of both chromophores with respect to indirect measurement of cortical activity via fNIRS (Obrig and Villringer 2003; Strangman et al. 2002). In general, HHb is described as the more valid parameter (Muthalib et al. 2015; Piper et al. 2014) since it is less contaminated by extra-cerebral processes (Kirilina et al. 2012). Taking this into account, results of the present study must be interpreted carefully since cycling tasks such as MICT are known to enhance systemic influence on fNIRS signal. Therefore, it seems reasonable to focus on the interpretation of HHb results and/or include additional analysis steps (e.g., SSR) to minimize potential confounding influences.

Concerning exercise-induced HHb concentration changes during MICT, our results revealed typical decreases at each intensity level, indicating increased activations in motor-related brain areas such as M1, PMC, SMA, IPL and SPL. A decrease in HHb has previously been reported by Kounalakis and Geladas (2012) during a constant load cycling task at a cycling rate of 80 rpm. Moreover, our findings indicate that HHb alterations might be dependent on exercise intensity. Regarding decreased HHb concentrations, these findings are reasonable and can be explained by previous studies demonstrating that more complex movements are associated with cortical activation increases in SMA and adjacent premotor areas (Orgogozo and Larsen 1979). Even though cortical processing during a rhythmic alternating activity such as cycling has mainly been investigated in prefrontal brain regions by means of fNIRS (Auger et al. 2016; Giles et al. 2014; Rupp et al. 2008), recent studies pointed to the important role of motor-related brain regions in cycling. For example, using PET, Christensen et al. (2000) observed an activation in M1 and SMA during active and passive cycling as two conditions of a rhythmic alternating activity in leg muscles. These findings were strengthened by subsequent fNIRS studies (Jung et al. 2015; Lin et al. 2012; Takehara et al. 2017), providing evidence for the important role of these brain regions in the planning, preparation and execution of complex movements (Leff et al. 2011). Hence, our findings and those of other groups demonstrate that there is a significant cerebral involvement in control of rhythmic locomotor movements in man. Beyond the fact that our results revealed a significant cerebral involvement during MICT, we also observed that HHb concentration changes might be modulated by increasing intensities (resistance) during cycling. This finding is further strengthened by previous studies demonstrating that higher cadences (MacIntosh et al. 2000) and intensities (Macdonald et al. 2008) during a cycling task are associated with a higher leg muscle activation. Hence, it is reasonable to assume that an increased recruitment of muscle fibers requires higher levels of neural resources in motor-related brain areas not only on a regional but also on a network level (Kenville et al. 2017). The fact that we observed intensity-dependent changes during cycling in a single brain region only (left PMC) does certainly not indicate that this area is exclusively responsible for modulating brain areas during increased workload during cycling. Even subcortical and/or cerebellar influences might potentially be involved in changing brain activation patterns during increased physical demands while executing movements (Maudrich et al. 2018; Sehm et al. 2016).

### No evidence for “neural efficiency” in athletes during MICT

We further hypothesized to find differential hemodynamic alterations as a response to exercise intensity between EA and ACP in accordance with the “neural efficiency” hypothesis (Dunst et al. 2014). We expected to find less brain activation in EA when compared to ACP at the same intensity level. However, we could not confirm this assumption, which contrasts with the “neural efficiency” hypothesis.

Recent studies have transferred the “neural efficiency” hypothesis into a context of motor tasks and expertise. There is evidence that extensive practice over a long period of time leads expert athletes to develop a focused and efficient organization of task-related neural networks (Milton et al. 2007) and that neural activity is reduced in motor experts (Del Percio et al. 2009). Several previous studies revealed that compared to novices/non-athletes, expert athletes show less brain activation during resting state (Babiloni et al. 2010) or performing cognitive/motor tasks such as strategic planning (Deeny et al. 2009), upright standing (Del Percio et al. 2009), balancing (Seidel et al. 2017), and wrist extension task (Del Percio et al. 2010). In a recent study, table tennis athletes and non-athletes performed a visuo-spatial task while cortical activation was examined using fMRI (Guo et al. 2017). Researchers found that athletes exhibited less brain activation than non-athletes in motor-related brain regions such as SMA and cerebellum, suggesting that long-term training prompts athletes to develop a focused and efficient organization of task-related neural networks. Contrarily, many other studies reported more or partly cortical activation in expert athletes compared to non-athletes, especially while observing sports videos (Aglioti et al. 2008; Orgs et al. 2008). During preparation or execution of a motor task, Del Percio et al. (2011) found higher alpha coherence values in parietal, temporal and occipital areas in elite pistol shooters using EEG. Findings of the present study also tend in this direction, indicating no differential effects in brain activation between EA and ACP. On the one hand, these findings might be due to task complexity of MICT, since cycling is a highly automated motor task not only for EA but also for ACP. Therefore, it seems reasonable to assume that larger effects of neural efficiency in athletes might be more likely to be observed in coordinatively more demanding and less familiar/automated movement patterns (Lyons et al. 2010; Woods et al. 2014). On the other hand, a recent study by Ludyga et al. (2016) suggests that in exercises with low coordinative demands (e.g., cycling) the level of cardiorespiratory fitness is of critical importance concerning neurophysiological correlates of neural efficiency (i.e., EEG signals). Authors showed that EA with higher *V*O_2_max values were able to complete submaximal cycling exercise with a lower level of brain cortical activity as compared to participants with a lower *V*O_2_max indicating enhanced neural efficiency in participants with high aerobic power. This inconsistency concerning the relation between neural efficiency and task complexity certainly deserves further investigation in future studies and is clearly beyond the scope of the current study.

### Study limitations

We used a multi-distance fNIRS approach to observe alterations in neurovascular coupling in motor-related brain areas during a cycling task in EA and ACP. To get a better understanding of neuronal correlates of complex motor tasks and potential effects of expertise, further studies using neurophysiological assessments of brain activation are needed. Our findings indicate that short-term exercise such as MICT seems to be less capable of inducing typical alterations in both chromophores Hb and HHb. Therefore, in future studies, duration of intensity levels as well as a clear distinction (e.g., larger intensities) between those levels need to be considered to observe typical and differential alterations. Additionally, fNIRS findings must be interpreted with caution, because, given our inter-optode distance, the penetration depth of infrared light is only ~ 1.5 cm (Pellicer and Bravo 2011; Perrey 2008). Hence, subcortical alterations obviously cannot be captured with this kind of imaging technique. Finally, we assessed brain activity predominantly over motor-related brain regions and did not use a whole-brain configuration to assess MICT-induced cortical alterations outside the human motor system. This seems particularly important since the vast majority of previous studies investigated exercise-dependent alterations in neurovascular coupling outside the human motor system focusing on prefrontal regions which are associated with cognitive domains but contribute indirectly to motor control (Giles et al. 2014; Rupp et al. 2008). For future studies, it should be mentioned that the validity of fNIRS during complex movements should be addressed in more detail, aiming to find task-dependent fNIRS modulations.

## Conclusion

In the present study, we used a multimodal approach that combined cardiorespiratory measurements and a multi-distance fNIRS measurement, including short-distance channels as a promising method to globally correct fNIRS signal during a complex motor task. We provide evidence that cycling at several intensity levels leads to hemodynamic response alterations within the human motor system measured as decreases in HHb. However, we could not find any differential effects in brain activations between EA and ACP, which is contrary to the “neural efficiency” hypothesis. For future studies, more knowledge on neural processing as well as whole-brain and systemic physiological augmented fNIRS measurements are required to get a holistic view, which seems to be of pivotal interest in studies aiming to assess neural activation during execution of complex movements in context of sport.
